# The 24.5-kb Left Variable Region Is Not a Determinant for African Swine Fever Virus to Replicate in Primary Porcine Alveolar Macrophages

**DOI:** 10.3390/v14102119

**Published:** 2022-09-25

**Authors:** Rui Luo, Tao Wang, Maowen Sun, Li Pan, Shujian Huang, Yun Sun, Hua-Ji Qiu

**Affiliations:** 1 State Key Laboratory of Veterinary Biotechnology, Harbin Veterinary Research Institute, Chinese Academy of Agricultural Sciences, 678 Haping Road, Harbin 150069, China; 2 School of Life Science Engineering, Foshan University, Foshan 528231, China

**Keywords:** African swine fever virus, multigene family genes, left variable region, cell tropism

## Abstract

African swine fever (ASF) is a widespread hemorrhagic and highly contagious infectious disease caused by African swine fever virus (ASFV), currently threatening the pig industry worldwide. Here, we demonstrated that the cell-adapted strain ASFV-P121 with a 24.5-kb deletion in the left variable region (LVR) lost the ability to replicate in primary porcine alveolar macrophages (PAMs). To explore whether this deletion determines the inability of ASFV-P121 replication in PAMs, a mutant virus (ASFV-ΔLVR) with the same LVR deletion as ASFV-P121 was constructed based on the wild-type ASFV HLJ/18 (ASFV-WT). However, the growth titer of ASFV-ΔLVR only reduced 10-fold compared with ASFV-WT in PAMs. Furthermore, we found that the large deletion of the LVR does not affect the formation of virus factories and virion morphogenesis. These findings reveal important implications for analyzing the molecular mechanism of ASFV cell tropism change.

## 1. Introduction

African swine fever (ASF) is a fatal hemorrhagic disease in domestic pigs and wild boars caused by African swine fever virus (ASFV), and the mortality of virulent ASFV infection is as high as 100%. ASF was first reported in Kenya in 1921 and is currently widely distributed across Eurasia, Africa, and Oceania [1]. ASF causes significant economic losses and threatens global food security. Only one live attenuated ASF vaccine has been approved for commercial use in Vietnam, but it has not been licensed in other countries [2]. The prevention and control of ASF mainly rely on culling the ASFV-infected pigs and strict biosecurity measures [3,4]. ASFV is a double-stranded DNA virus with an average diameter of about 250 nm, encoding approximately 200 proteins. The genome of different ASFV isolates ranges from 170 to 194 kb. The ASFV genome consists of the conserved central region (CCR), the left variable region (LVR) of about 40.5 kb, and the right variable region (RVR) of about 20 kb [5,6]. Most multigene family (MGF) genes of LVR play key roles in viral replication, immune escape, and pathogenicity [7]. For example, MGF360-9L is an important virulence factor associated with the ASFV by antagonizing the JAK/STAT-signaling pathway [8].

Monocytes and macrophages are the primary target cells of ASFV infection. Primary porcine alveolar macrophages (PAMs) have been widely used to culture ASFV and to study virus–host interactions [9]. However, the preparation of PAMs is time-consuming and expensive. Moreover, using PAMs for live attenuated vaccines (LAV) production is unideal [10]. To address the above-mentioned problems, some ASFV isolates have been adapted to replicate in cell lines by successive passages [11,12,13,14]. Most cell-adapted ASFVs are attenuated in pigs; nevertheless, they tend to lose immunogenicity and do not confer immune protection [13]. During the passage of ASFV in the cell lines, large deletions in the variable regions, as well as scattered point mutations, occur in the genome [11,13,15]. For example, the Vero cell-adapted ASFV strain BA71V has lost 18 MGF genes in the LVR [11]. The adaptation of ASFV E70 to monkey kidney cells (MS) was accompanied by an approximately 20-kb deletion in the variable region of the genome [14]. A large deletion in the LVR has also been found in the genomes of naturally attenuated ASFV isolates OURT88/3 and NH/P68 [15]. Our previous study indicated that the ASFV HLJ/18 strain could replicate efficiently after adaptation to HEK293T cells. Interestingly, all of the above cell-adapted ASFV strains lost the ability to replicate in target cells [13,16]. However, it is presently unknown whether the genes of LVR determine the ability of ASFV to replicate in PAMs.

To investigate whether the deletion of the genomic region in LVR renders cell-adapted ASFV strains unable to replicate in PAMs, we constructed a mutant virus ASFV-ΔLVR with major LVR deletion based on the ASFV HLJ/18 strain. Replication kinetics of ASFV-ΔLVR showed that the deletion of the 24.5-kb LVR was not a determinant for the inability of ASFV-P121 to replicate in PAMs. Viral gene mutations might also contribute to the altered phenotype of ASFV-P121.

## 2. Materials and Methods

### 2.1. Cells and Viruses

PAMs were cultured in RPMI 1640 medium with L-glutamine (catalog no. C11875500BT; Gibco) supplemented with 10% fetal bovine serum (FBS) (catalog no. 10091148; Gibco) and 2% antibiotics–antimycotics (catalog no. 15140122; Gibco) at 37 °C with 5% CO_2_ [17,18]. The ASFV HLJ/18 strain (ASFV-WT) (GenBank accession number MK333180.1) was propagated and titrated on PAMs [19]. The cell-adapted virus ASFV-P121 was propagated as described previously [13]. All the experiments involving the live ASFV in this study were performed in the biosafety level 3 facility of Harbin Veterinary Research Institute (HVRI) of the Chinese Academy of Agricultural Sciences (CAAS) and approved by the Ministry of Agriculture and Rural Affairs, China.

### 2.2. Hemadsorption Assay (HAD)

PAMs (2 × 10^6^ cells) seeded in 96-well cell culture plates were infected with five multiplicities of infection (MOI) of ASFV. Fresh swine red blood cells (2 × 10^6^ cells/well) were added to each well at 48 h post-infection (hpi) and incubated for three days. The rosettes were observed under a microscope [20,21].

### 2.3. Western Blotting

PAMs were infected with ASFV-P121 and ASFV-WT at an MOI of 5. The cells were washed with PBS and lysed in RIPA buffer supplemented with protease inhibitors at 12, 24, and 48 hpi. The cell lysates were resolved by electrophoresis on a 12.5% SDS-PAGE gel and transferred to a polyvinylidene fluoride membrane. The membrane was blocked using 5% skim milk in Tris-buffered saline with 0.05% Tween 20 (TBST) and incubated with appropriate primary and secondary antibodies as described previously [22].

### 2.4. Virus Replication Kinetics

PAMs were infected with ASFV at 0.01 MOI in 24-well cell culture plates. After 2-h incubation at 37 °C with 5% CO_2_, the inoculum was removed, and the cells were washed with PBS. The virus was harvested at 2, 12, 24, 72, 120, and 168 hpi. Viral genome copies and titers were determined as described previously [13,19,23].

### 2.5. Generation of a Mutant ASFV with the 24.5-kb LVR Deleted

A mutant virus (ASFV-∆LVR) with a deletion of LVR was developed by homologous recombination of the ASFV-WT genome and the transfer vector pOK12-p72EGFP-ΔLVR [24]. The transfer vector harbors the *p72* promoter-controlled *EGFP* gene (*p72*EGFP) and the left and right arms spanning the ASFV genomic positions 7273–8131 and 33070–34257, respectively. The sequence of the genomic positions 8436 to 32970 was replaced with *p72*EGFP. PAMs seeded in 6-well plates were incubated for 12 h at 37 °C and transfected with 3 μg of pOK12-p72EGFP-ΔLVR using X-tremeGENE HP for 24 h and then infected with ASFV-WT (MOI = 3). The recombinant mutant virus ASFV-ΔLVR was purified by multiple rounds of limiting dilution based on the expression of the *EGFP* gene. The integrity and fidelity of the modified region were confirmed by PCR and DNA sequencing. The primers used in this study are listed in Table 1.

### 2.6. Next-Generation Sequencing (NGS)

The viral genomic DNA was extracted from the ASFV-∆LVR-infected PAMs by the QIAamp blood mini kit (catalog no. 51104; Qiagen, Hilden, Germany). The full-length sequence of the ASFV-∆LVR genome was determined by NGS as described previously [13,25].

### 2.7. Transmission Electron Microscopy (TEM)

PAMs were seeded in 24-well cell culture plates for 12 h and then infected with ASFV-WT or ASFV-∆LVR at an MOI of 5 and fixed with 2% glutaraldehyde at 24 hpi. The fixed cells were prepared for TEM following the protocols described previously [26].

### 2.8. Statistical Analysis

All data were statistically analyzed by an unpaired two-tailed Student’s *t*-test to determine statistical significance and performed in GraphPad Prism 8.0. The data are presented as the mean ± SD of three independent experiments. *p* ≥ 0.05 means the difference is not significant (ns); *p* < 0.05 was considered statistically significant (* *p* < 0.05; ** *p* < 0.01; *** *p* < 0.001).

## 3. Results and Discussion

### 3.1. The Cell-Adapted ASFV with Varying LVR MGF Deletions Lost the Ability to Replicate in PAMs

**Figure 1:**
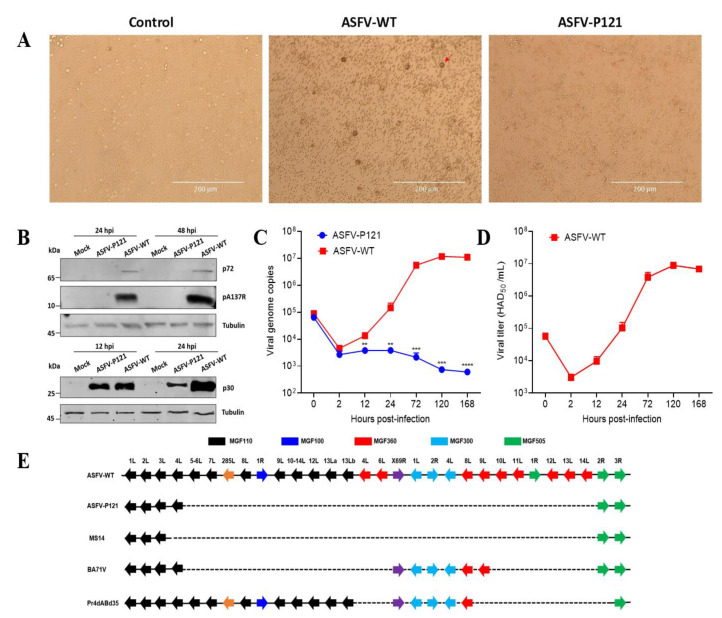
Cell-adapted ASFV lost the ability to replicate in PAMs. (**A**) Hemadsorption assay of the PAMs infected with ASFV-P121 or ASFV-WT (MOI = 5). Pig erythrocytes were added to the ASFV-infected PAMs. The presence of rosettes was checked at 72 hpi (scale bars, 400 µm). The red arrow indicated a rosette of red blood cells. (**B**) Analysis of ASFV protein expression in PAMs. PAMs were infected with ASFV-P121 or ASFV-WT (MOI = 5), and the viral proteins were detected using the antibodies against the p72, A137R, and p30 proteins. (**C**,**D**) In vitro growth kinetics of ASFV-P121 or ASFV-WT in PAMs. PAMs were infected with ASFV-P121 or ASFV-WT (MOI = 0.01). The cell cultures were obtained at the indicated time points post-infection, and ASFV genome copies (**C**) and titers (**D**) were determined. The data are presented as the mean ± SD of three independent experiments. (** *p* < 0.01; *** *p* < 0.001; **** *p* < 0.0001). (**E**) Schematic representation of the *MGF* genes deletion observed at the left variable region in the genome of the cell-adapted ASFV mutants.

Recently, we developed an HEK293T cell-adapted ASFV (ASFV-P121) [13]. To determine whether ASFV-P121 loses the ability to replicate in PAMs, as do the BA71V, MS14, and ASFV-G/VP110 strains [16,27], we performed a hemadsorption (HAD) assay and no erythrocyte adsorption was observed in the PAMs infected with ASFV-P121 (Figure 1A). Next, the expression of the viral early protein p30 and the late proteins p72 and A137R was examined to evaluate whether ASFV-P121 can replicate in PAMs. As shown in Figure 1B, in contrast with ASFV-WT, the p72 and A137R proteins were not detectable, but the early protein p30 was detected in the ASFV-P121-infected PAMs. Moreover, the viral genome copies (Figure 1C) and titers (Figure 1D) gradually increased and peaked at 120 hpi in the ASFV-WT-infected PAMs. However, no replication of the viral genome (Figure 1C) or infectious virus (Figure 1D) was detected in the ASFV-P121-infected PAMs. These data indicate that ASFV-P121 could enter PAMs and initiate early gene expression but underwent abortive replication.

Subsequently, we performed genome sequence alignment to screen the possible determinant of the cell-adapted ASFV strains (e.g., ASFV-P121, MS14, and BA71V) that cannot replicate in PAMs. We found that the most significant variation was the deletion of varying MGF genes in the LVR of the cell-adapted ASFV (Figure 1E). The LVR deletion in the ASFV-P121 genome is up to 24.5 kb, covering many MGF genes, including MGF110, MGF300, MGF505, and MGF360 [13]. Moreover, the varying MGF deletions within the LVR were also reported in the naturally attenuated ASFV, including OURT/88/3 and NHP68 [28,29]. Therefore, we hypothesize that the LVR MGF genes-encoded proteins may be the main factors affecting the replication of ASFV in PAMs.

### 3.2. The 24.5-kb LVR Is Non-Essential for ASFV Replication and Virion Morphogenesis in PAMs

**Figure 2:**
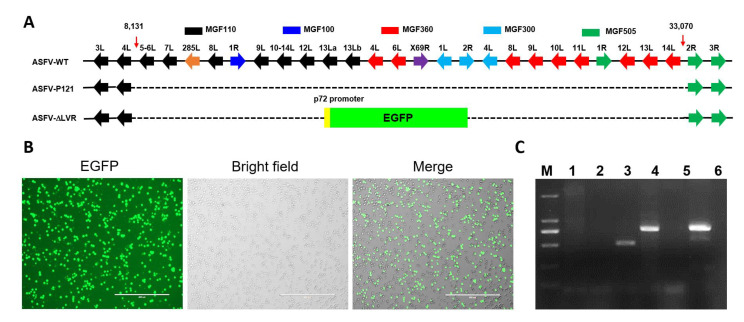
Construction and characterization of the LVR-deleting ASFV mutant. (**A**) Schematic diagram of ASFV-∆LVR construction. The left variable region (LVR) MGF genes were replaced with the p72EGFP expression cassette via homologous recombination. (**B**) Purified ASFV-∆LVR expressing EGFP. (**C**) PCR analysis of ASFV-∆LVR stock. Line 1: ASFV-∆LVR genome used as a template to amplify the *MGF300-1L* gene; Line 2: negative control with ddH_2_O as a template; Line 3: ASFV-WT genome used as a template to amplify the *MGF300-1L* gene; Line 4: ASFV-∆LVR genome used as a template to amplify EGFP and left homology arm; Line 5: negative control with ddH_2_O as a template; Line 6: positive control with the transfer vector pOK12-p72EGFP-∆LVR as a template.

**Figure 3:**
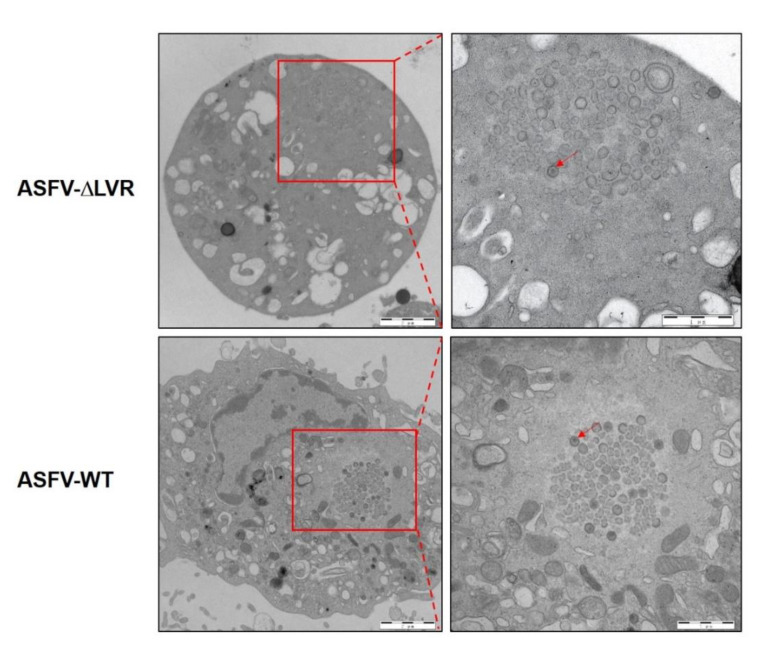
Morphology of virus factories in the ASFV-infected PAMs. PAMs were infected with ASFV-WT or ASFV-∆LVR (MOI = 5) and fixed with 2% glutaraldehyde at 24 hpi. The fixed cells were analyzed by transmission electron microscopy.

To validate the above hypothesis, a mutant ASFV with the 24.5-kb LVR deleted (ASFV-ΔLVR) was generated (Figure 2A). The ASFV-ΔLVR was purified after 15 rounds of limited dilution based on the EGFP expression (Figure 2B). The accuracy of the genomic modifications introduced into the ASFV-ΔLVR was confirmed by PCR and Sanger’s sequencing (Figure 2C). Furthermore, de novo sequencing of the ASFV-ΔLVR genome was performed based on NGS. The total reads were 11,516,922, of which 2.04% aligned to the ASFV-WT genome. The mean coverage of reads corresponding to the ASFV-ΔLVR was 175.53. De novo assembly of the ASFV-ΔLVR genome was performed as described in Materials and Methods. The complete ASFV-ΔLVR genome was 165,588 bp with a deletion of 24,537 bp in LVR. No other significant genomic differences were detected in the ASFV-ΔLVR genome. Further analysis showed that ASFV-ΔLVR has the ability of erythrocyte adsorption (data not shown) and can replicate in PAMs. The deleted 24.5-kb LVR may contain some structural proteins involved in ASFV morphogenesis. Therefore, the TEM was used to analyze the ultrastructure of ASFV-ΔLVR and ASFV-WT. However, no differences in the viral factories and virion morphogenesis were observed between ASFV-ΔLVR and ASFV-WT (Figure 3). These results indicate that the 24.5-kb LVR is non-essential for ASFV replication and virion morphogenesis in PAMs.

### 3.3. The Growth of the ASFV Mutant Lacking the 24.5-kb LVR Was Decreased in PAMs

**Figure 4:**
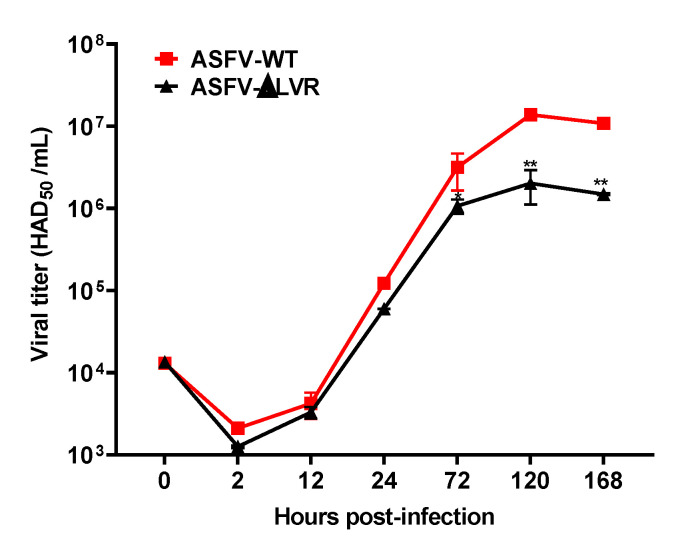
The viral growth of the LVR-deleted ASFV mutant in PAMs. PAMs were infected with ASFV-WT or ASFV-∆LVR (MOI = 0.01), and virus titers yields were determined at the indicated –time points post-infection. The data are presented as the mean ± SD of three independent experiments (* *p* < 0.05; ** *p* < 0.01).

To determine the effects of the deletion of LVR on replication of ASFV in PAMs, the replication kinetics of ASFV-ΔLVR were compared with that of ASFV-WT. Interestingly, ASFV-ΔLVR could replicate in PAMs but with a titer about 10-fold lower than that of the parental strain ASFV-WT (Figure 4). It is possible that the deleted LVR may contain a key *MGF* gene for ASFV to replicate in PAMs. However, it is difficult to speculate which *MGF* gene is critical for the replication of ASFV in PAMs, since the roles of the individual MGF genes, especially in MGF300 and MGF110, remain poorly understood [30].

A previous study has shown that the mutant ASFV Pr4dABd35 with MGF505 (*1R* and *2R*) and MGF360 (*4L*, *6L*, *9L*, *10L*, *11L*, *12L*, and *14L*) deletion significantly reduced the viral growth in PAMs [16]. In contrast, the *MGF505-2R* gene is not deleted in ASFV-ΔLVR, suggesting that this gene may play an important role for ASFV to replicate in PAMs. Although ASFV-ΔLVR did not rescue the ASFV-P121 phenotype in PAMs, the effects of mutations outside the LVR *MGF* genes in the ASFV-P121 genome cannot be excluded. Whether the point mutations observed in the ASFV-P121 genome, including *I7L*, *I8L*, *I9L*, *I10L*, *MGF100-3L*, and *E199L* genes, affect the replication of ASFV-P121 in PAM cannot be ruled out [13]. For example, a T-to-A mutation in the *E199L* gene resulted in an N131I substitution in the protein, which is required for membrane fusion and penetration [13]. Thus, we speculate that the inability of ASFV-P121 to replicate in PAMs may result from the combined effects of the large LVR deletion and dispersed point mutations.

The *MGF360* (*9L*, *10L*, *11L*, *12L*, *13L*, and *14L*) and *MGF505* (*1R*, *2R*, and *3R*) genes are associated with viral pathogenesis and virulence in pigs but not with viral replication in PAMs [7]. Recently, the recombinant viruses ASFV-Δ9L and ASFV-Δ360-9L, which were constructed by deleting the *MGF110-9L* or *MGF360-9L* gene, showed reduced replication in PAMs in comparison with the parent strain and exhibited attenuated virulence in pigs upon infection with a low dose [8]. We found that the above-mentioned *MGF* genes, including *MGF360* (*10L*, *11L*, *12L*, *13L*, and *14L*), *MGF505-1R*, *MGF360-9L*, and *MGF110-9L*, were deleted in the genome of ASFV-ΔLVR, indicating that ASFV-ΔLVR might exhibit an attenuated phenotype in pigs. Future studies are warranted to evaluate the pathogenicity and immunogenicity of ASFV-ΔLVR in vivo.

In summary, the 24.5-kb left variable region is not a determinant for the inability of the HEK293T cell-adapted ASFV to replicate in PAMs.

## Figures and Tables

**Table 1:** Primers were used in this study.

Primers	Sequences (5′→3′)	Description
LA-F	GGTACCCGGGAGCTCGAATTCTAAAAGCCTTACAGATCATCC	Amplification of left homology arm
LA-R	CATCTCTCACGAGATCGTGACTGAAAACAGCTACTTGACAAC	
72EGFP-F	GTTGTCAAGTAGCTGTTTTCAGTCACGATCTCGTGAGAGATG	Amplification of p72EGFP
72EGFP-R	ATGGGCTTTATAGTCCTTTGTCCTGTGAGATCATGGCAGCT	
RA-F	AGCTGCCATGATCTCACAGGACAAAGGACTATAAAGCCCAT	Amplification of right homology arm
RA-R	GTCTGCAGAAGCTTCGAATTCTCTCATTTTCTGTATAGCCAT	
PF	AAATCCTGTTAAATAGGCAAA	Amplification of the *MGF300-1L* gene
PR	AGTAAAATATTAGAGCGTCTG	
RF	GATCCGCCACAACATCGAG	Amplification of the *EGFP* and right homology arm sequence
RR	TTTTCAAACGTATTCGCGTCT	

## Data Availability

The sequencing data have been deposited with the National Microbiology Data Center under the Bioproject ID: NMDC10017733.
